# Generation and application of TGFβ-educated human Vγ9Vδ2 T cells

**DOI:** 10.1016/j.xpro.2022.101319

**Published:** 2022-04-18

**Authors:** Ana C. Parente-Pereira, Richard E. Beatson, David M. Davies, Caroline Hull, Lynsey M. Whilding, Joanna C. Porter, John Maher

**Affiliations:** 1 School of Cancer and Pharmaceutical Sciences, King’s College London, Faculty of Life Sciences and Medicine, Guy’s Campus, London SE1 9RT, UK; 2 UCL Respiratory, University College London, Bloomsbury Campus, London WC1E 6AE, UK; 3 Leucid Bio Ltd., Guy’s Hospital, Great Maze Pond, London SE1 9RT, UK; 4 Department of Respiratory Medicine, University College London Hospitals, Euston Road, London NW1 2BU, UK; 5 Department of Immunology, Eastbourne Hospital, Kings Drive, Eastbourne, East Sussex BN21 2UD, UK

**Keywords:** Cell Biology, Cell culture, Flow Cytometry/Mass Cytometry, Cell-based Assays, Cancer, Health Sciences, Immunology, Model Organisms, Molecular Biology, Biotechnology and bioengineering

## Abstract

Clinical trials that tested the antitumor activity of γδ T cells have been mostly unsuccessful. To address this, we expanded human Vγ9Vδ2 T cells in TGFβ1, a cytokine which enhances their viability, trafficking properties, and intrinsic antitumor activity. This protocol summarizes the production and *in vitro* functional characterization of TGFβ1 educated human Vγ9Vδ2 cells and highlights their compatibility with chimeric antigen receptor (CAR) engineering. We also describe *in vivo* testing of the antitumor activity of these CAR T cells in mice.

For complete details on the use and execution of this protocol, please refer to Beatson et al. (2021).

## Highlights

• TGFβ promotes viability and retards differentiation of Vγ9Vδ2 cells
• TGFβ-educated Vγ9Vδ2 cells achieve greater invasion and anticancer activity
• Specificity of these cells can be reprogrammed using chimeric antigen receptors

## Graphical abstract

**Figure:**
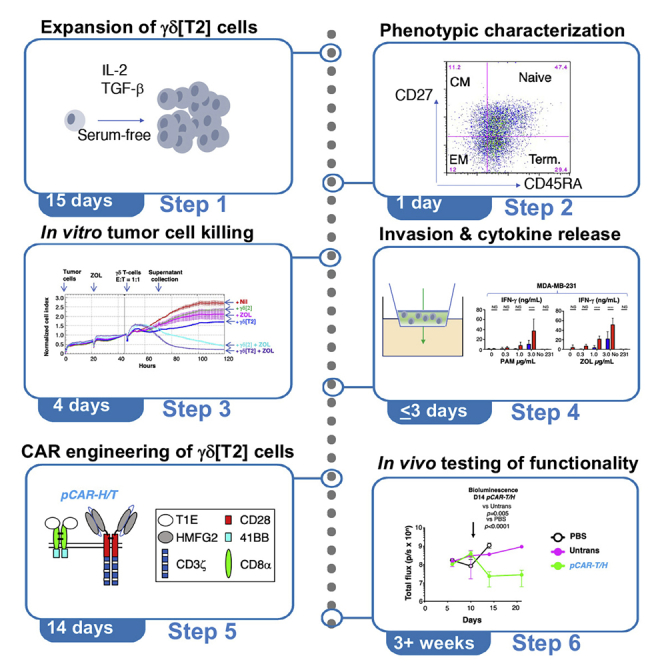

## Before you begin

### Experimental consideration

1. γδ T cells play a key role in immune surveillance for malignant transformation (Kabelitz et al., 2020). Moreover, the presence of intra-tumoral γδ T cells is strongly predictive of improved prognosis across a range of cancer types (Gentles et al., 2015). The dominant circulating γδ T cell subtype (δ2 subtype) expresses a Vγ9Vδ2 T cell receptor that detects phosphoantigen intermediates of mevalonate metabolism, a metabolic pathway that is upregulated in transformed cells. These cells can be selectively activated using aminobisphosphonate drugs such as zoledronic acid [ZOL]. Non δ2 γδ T cells are also present in the circulation, albeit at lower levels. These cells also possess intrinsic anti-tumor activity. In order to activate both γδ T cell subtypes, an immobilized pan-γδ T cell receptor antibody may be used. Given the intrinsic anti-tumor activity of both δ2 and non δ2 γδ T cells and the fact that neither subtype causes graft versus host disease, these cells are the subject of great interest as a potential off-the-shelf approach to adoptive cancer immunotherapy.
2. Recombinant human TGFβ1 available from different suppliers varies in its bioactivity. We use TGFβ1 from Bio-Techne and have validated its activity in house using a TGFβ1 reporter system (Abe et al., 1994). We have tested products from other manufacturers, although none have performed as robustly.
3. We have used either ZOL or an immobilized anti-γδ TCR antibody (11F2 or B1 clones) to elicit the activation of circulating γδ T cells, enabling their subsequent cytokine-driven expansion. Using the immobilized anti-γδ TCR antibody method, non-Vδ2 cells are also expanded. We have not tested other antibodies for the expansion of these cells (e.g., anti-Vγ9 antibodies). Moreover, we have not tested this method for the expansion of tissue-resident γδ T cells or using other isoforms of TGFβ.
4. Extensive optimization of the system was carried out using different TGFβ1 concentrations, dosing regimens and media. Importantly, these effects of TGFβ1 could be recapitulated in media containing human serum, and in other serum-free media. However, the serum-free protocols described in this paper were found to be optimal.
5. A variety of different starting materials were trialed as a source of Vγ9Vδ2 cells with freshly isolated whole blood providing the most consistent and optimal expansion numbers. Other starting materials tested were leukocytes cones, whole blood from the National Blood and Tissue Service (Tooting, London) and frozen PBMCs.
6. Except for centrifugation and the blood draw, all steps in this protocol involving cell culture are performed in a laminar flow hood using aseptic technique.

### Institutional permissions

All *in vivo* experimentation must comply with local regulatory requirements. In this case, experimental work was compliant with U.K. Home Office guidelines, as specified in project license number P23115EBF. In addition, all work was approved by the King’s College London animal welfare and ethical review body (AWERB).

## Key resources table

**Table:** 

REAGENT or RESOURCE	SOURCE	IDENTIFIER
**Antibodies**		

Anti-human/primate EGF biotinylated – 1/150 dilution	R&D Systems	Cat# BAF236; RRID: AB_356307
CD27 – PE (M-T271) – 1/100 dilution	BioLegend UK	Cat# 356405, RRID: AB_2561824
CD45RA – BV605/ APC (HI100) – 1/100 dilution	BioLegend UK	Cat# 304150, RRID: AB_2564158
PE Goat anti-mouse IgG – 1/100 dilution	BioLegend UK	Cat# 405307
Streptavidin PE (SA-PE) - – 1/150 dilution	BioLegend UK	Cat# 405203
TCR Pan γδ purified (11F2) – coat plates with 0.8 mg/mL	BD Biosciences	Cat# 347900, RRID: AB_400356
TCR Pan γδ - FITC (B1) – 1/100 dilution	BD Biosciences	Cat# 559878, RRID: AB_397353
Mouse IgG1 FITC isotype – 1/100 dilution	BD Biosciences	Cat# 345815, RRID: AB_2868833
Ultra-LEAF CD11a blocking antibody – block with 10 μg/mL	BioLegend UK	Cat# 301233, RRID: AB_2832576
Ultra-LEAF Mouse IgG1 LEAF – block with 10 μg/mL	BioLegend UK	Cat# 400166, No RRID available
Ultra-LEAF Pan γδ TCR (B1) - coat plates with 0.8 mg/mL	BioLegend UK	Cat# 331235, RRID: AB_2814201

**Bacterial and virus strains**		

DH5α *E. coli*	New England Biolabs	Cat# c2987h
SFG retroviral vector	Dr Michel Sadelain, Memorial Sloan Kettering Cancer Center	N/A

**Biological samples**		

Human blood	Healthy volunteers, male and female, aged 18–65 years	Approved by the West of Scotland Research Ethics Committee 3 (REC reference 18/WS/0047)

**Chemicals, peptides, and recombinant proteins**		

Antibiotic antimycotic (100×)	Thermo Fisher Scientific	Cat# 15240062
Bovine serum albumin	Sigma-Aldrich	Cat# A9418
D-luciferin	Cambridge Bioscience	Cat# B3000-1G
DMEM medium	Lonza	Cat# BE12-604Q
Disodium edetate (EDTA)	Sigma-Aldrich	Cat# BP1224
Engelbreth-Holm-Swarm (EHS) basement membrane extract (BME)	Sigma-Aldrich	Cat# 126-2.5
Fetal bovine serum	Gibco	Cat# 26140079
Ficoll®-Paque PLUS	GE Healthcare	Cat# GE17-1440-02
GeneJuice® transfection reagent	Sigma-Aldrich	Cat# 70967-3
Isoflurane	Sigma-Aldrich	Cat# 792632
L-Glutamine solution	Sigma-Aldrich	G7513-100ML
Luciferin	Regis Technologies	Cat# 115144-35-9
MTT	Apollo Scientific	Cat# BID2165
Pamidronate sodium	Pfizer Ltd.	N/A
PBS	Sigma-Aldrich	D8537
Proleukin (aldesleukin), human recombinant interleukin (IL)-2	Clinigen Group	N/A
Recombinant Human TGF-β1	Bio-Techne	Cat# 240-B
RetroNectin® recombinant human fibronectin fragment	Takara Bio	Cat# T100B
Sodium Citrate	USP	1613859
TexMACS^TM^ medium	Miltenyi Biotec	130-097-196
Trypan Blue	Sigma-Aldrich	Cat# T8154
Zometa	Novartis	N/A

**Critical commercial assays**		

Human uncoated IL-2 ELISA kit	Life Technologies Ltd	Cat# 88-7025-88
Human IFN-gamma DUOset ELISA	Bio-Techne	Cat# DY285B

**Experimental models: Cell lines**		

BT-20	Breast Cancer Now Research Unit, King’s College London	ATCC Cat# HTB-19 RRID CVCL_0178
BxPC-3	Barts Cancer Institute, Queen Mary University of London	ATCC Cat# CRL-1687 RRID CVCL_0186
HEK293T	ATCC	Cat# CRL-3216 RRID: CVCL_0063
MDA-MB-231	Breast Cancer Now Research Unit, King’s College London	ATCC Cat# HTB-26 RRID: CVCL_0062
MDA-MB-468	Breast Cancer Now Research Unit, King’s College London	ATCC Cat# HTB-132 RRID CVCL_0419
Ovsaho	Japanese Collection of Research Bioresources Cell Bank	Cat# JCRB1046 RRID CVCL_3114
PG13	European Collection of Authenticated Cell Culture	Cat# 95110215 RRID CVCL_4273
TOV-21G	Prof Sadaf Ghaem-Maghami, Imperial College London	ATCC Cat# CRL-11730 RRID: CVCL_3613

**Experimental models: Organisms/strains**		

NOD.Cg-Prkdc^SCID^ Il2rg^tm1Wjl^/SzJ (NSG) mice, male and female, aged 6–10 weeks	Charles River	Strain code: 614

**Recombinant DNA**		

PeqPam plasmid	Gift of Dr M Pule, University College London	N/A
*pCAR-H/T*	CAR Mechanics Group, King’s College London	(Muliaditan et al., 2021)
RDF plasmid	Gift of Prof M Collins, University College London	N/A

**Software and algorithms**		

FlowJo v.10 Software	Tree Star	https://www.flowjo.com/
Gene Designer	DNA2.0	https://www.atum.bio/resources/tools/gene-designer
Prism 9	GraphPad	https://www.graphpad.com/scientificsoftware/prism
SnapGene	GSL Biotech	https://www.snapgene.com/

**Other**		

Adhesive PCR Plate Seals	Thermo Fisher Scientific	Cat# AB0558
MicroAmp™ Optical 96-Well Reaction Plate	Thermo Fisher Scientific	Cat# n8010560
RTCA E-plates	Agilent	Cat# 300601010
Sodium heparin blood collection tubes	BD Biosciences	Cat# 367874
ThinCert^TM^ inserts for 24 well plates (6.5 mm, 3 μm pore size)	Greiner Bio-One	Cat# 662631
xCELLigence RTCA MP	Agilent	N/A
BD LSRFortessa^TM^ X-20	BD Biosciences	N/A

## Materials and equipment

**Table:** TexMACS^TM^+ medium

Reagent	Final concentration	Amount
TexMACS^TM^ medium	×1	490 mL
L-Glutamine (×100)	2 mM	5 mL
Antibiotic Antimycotic (×100)	×1	5 mL
**Total**	N/A	500 mL

***Note:*** Store TexMACS^TM^+ medium at 4°C for up to 2 weeks.

**Table:** D10 medium

Reagent	Final concentration	Amount
DMEM medium	×1	440 mL
Fetal bovine serum	10%	50 mL
L-Glutamine (×100)	2 mM	5 mL
Antibiotic Antimycotic (×100)	×1	5 mL
**Total**	N/A	500 mL

***Note:*** Store D10 medium at 4°C for up to 2 weeks.

**Table:** FACS buffer

Reagent	Final concentration	Amount
PBS	×1	99 mL
Disodium edetate (0.5 M stock)	0.5 mM	100 μL
Fetal bovine serum	1%	1 mL
**Total**	N/A	100 mL

***Note:*** Store Disodium edetate (0.5 M stock) for up to 1 year at 4°C.

***Note:*** Prepare FACS buffer fresh before use.

**Table:** Equipment

Flow cytometer	Analytical software	Version
BD LSRFortessa^TM^ X-20	FlowJo^TM^	V10.8.1

## Step-by-step method details

**Timing: 15 or more days, depending on expansion methodology used**

Expansion of activated Vγ9Vδ2 T cells in the presence of TGFβ1.

This step describes methods used to activate and expand Vγ9Vδ2 T cells over a 2 week period in serum-free medium supplemented with IL-2 and TGF-β1.

***Note:*** Cells expanded using the combination of IL-2 and TGF-β1 are designated γδ[T2] cells. If cells are expanded in the same way but using IL-2 alone, they are referred to as γδ[2] cells.

***Note:*** Our RNA sequencing data suggests that cells are not phenotypically mature at day 8 (Beatson et al., 2021). We therefore recommend completing the 15 day culture period.

Day -1.

1. *Alternative (antibody activation method only)*: Coat 24, 12 or 6 well plates (depending on the numbers of PBMCs expected) overnight (12–16 h) at 4°C with 0.8 mg/mL pan γδ TCR antibody (11F2 or B1 clones) contained in a volume of 1 mL, 2 mL or 3 mL PBS, respectively.

**CRITICAL:** We have not tested other pan γδ TCR antibodies for their ability to stimulate the activation of these cells.

Day 0.

2. Withdraw blood (typically 45 mL) from a consenting healthy volunteer and mix with 5 mL sodium citrate in pre-prepared 50 mL Falcon tubes (stored up to 6 months at 4°C).

***Note:*** Alternatively, blood can be collected into sodium heparin blood collection tubes.

3. Gently mix blood with an equal volume of PBS (1:1 ratio).
4. Gently layer the blood/PBS mix (using the gravity setting on a pipette controller) on top of 15 mL Ficoll Paque Plus in 50 mL Falcon tubes.
5. Centrifuge the 50 mL tubes at 750 × *g* for 30 min at 20°C (acceleration and brake set to 0) to separate the PBMC cell fraction.
6. Transfer the PBMC layer, seen as an interface between the plasma and the Ficoll layer, using a sterile Pasteur pipette to a fresh 50 mL Falcon tube.

***Note:*** After centrifugation, the layer above the PBMC fraction which contains plasma can be carefully removed using a Pasteur pipette to make the PBMC layer easier to harvest.

7. In the 50 mL tube containing the PBMCs, add PBS to a final volume of 50 mL and centrifuge at 500 × *g* for 5 min. The acceleration and brake can be set back to 9.
8. Aspirate and discard the supernatant leaving the pelleted cells.
9. If more than one tube has been used (due to the volume size), resuspend the pellets in TexMACS^TM^+ and combine in a single tube. Bring the cells up to a final volume of 50 mL of PBS for a second wash.
10. Centrifuge at 500 × *g* for 5 min.
11. Aspirate and discard the supernatant.
12. Resuspend the cell pellet an appropriate volume of TexMACS^TM^+ medium (see materials and equipment Table above; usually 10 mL per 45 mL blood).

**CRITICAL:** We have not tested other serum free media for their ability to support the expansion of γδ T-cells.

13. Count live cells using trypan blue staining and a hemocytometer. Resuspend cells at 3 × 10^6^/mL.

***Note:*** Samples are normally diluted 1 in 10 with PBS prior to cell counting.

14. *Alternative (antibody activation method only – to expand both δ2 and non-δ2 γδ T cells)*: Aspirate the antibody-containing PBS solution from plates set up in step 1. Wash each well once with PBS. Plate PBMC immediately at a density of 3 × 10^6^ cells/mL in 4 mL TexMACS^TM^+ medium per well of a 6 well plate. Add recombinant IL-2 (100 U/mL) and recombinant TGF-β1 (5 ng/mL). After 72 h, remove the cells are removed from this plate.

OR

15. *Alternative (zoledronic acid method only – to expand δ2 γδ T cells)*: Plate PBMC at a density of 3 × 10^6^ cells/mL in TexMACS^TM^+ medium. Add zoledronic acid (1 mg/mL), together with recombinant IL-2 (100 U/mL) and recombinant TGF-β1 (5 ng/mL).

***Note:*** We do not observe consistent differences in total yield of γδ T cells achieved using the two methods although the relative proportion of non δ2 γδ T cells that expand is greater using the antibody method.

Days 1–14.

16. Monitor cell density by microscopy and cell counting every 2–3 days.
  a. If cell density exceeds 1 × 10^6^ cells/mL, add 100% TexMACS^TM^+ medium followed by recombinant IL-2 (100 U/mL) and recombinant TGF-β1 (5 ng/mL).
  b. If cell density is below this level, add cytokines alone.

***Note:*** Cytokine addition is corrected to the total volume of medium present. Where necessary, split and expand cultures into additional wells on the chosen plate type, and thereafter into T75 flasks.

Day 15.

**Figure 1:**
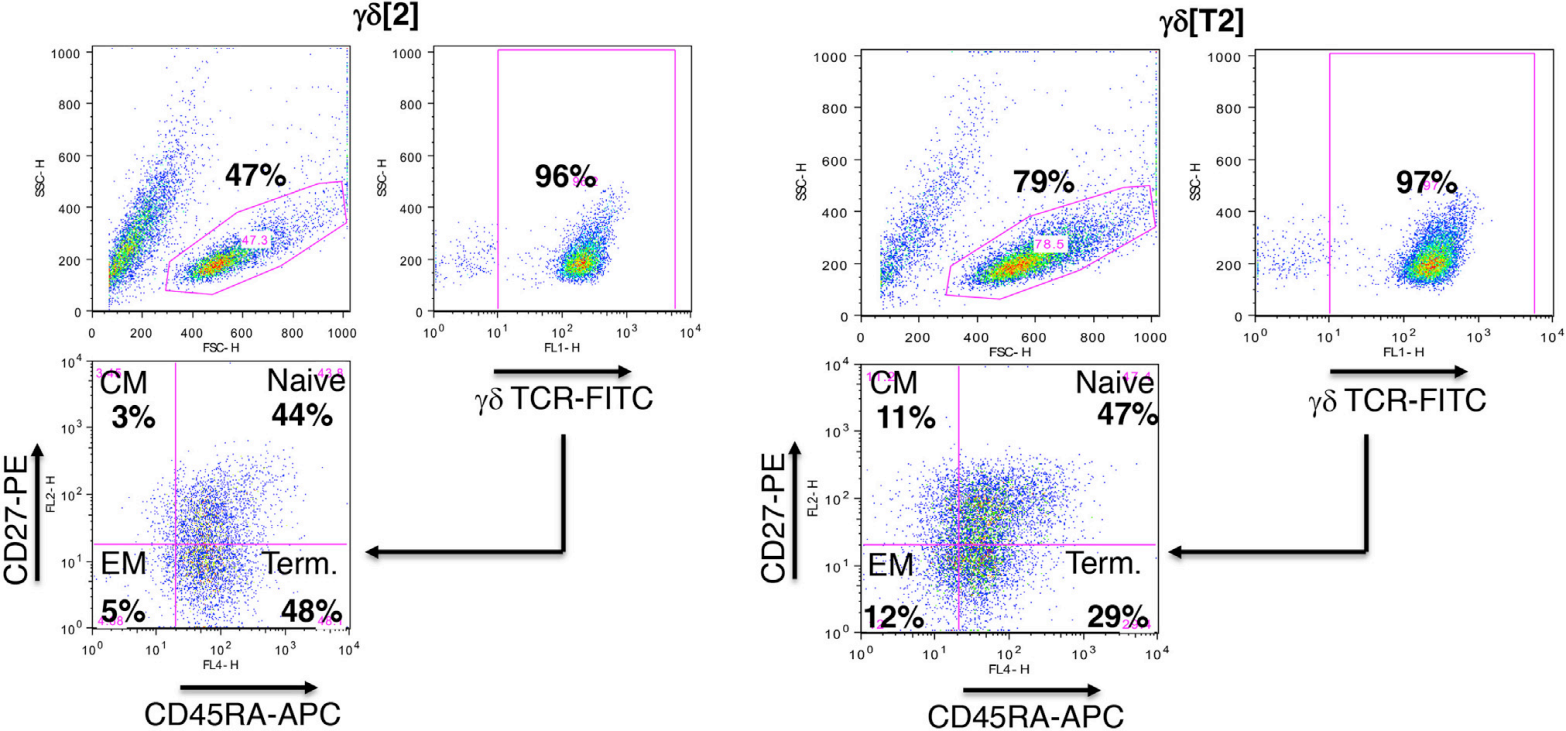
Assessment of differentiation state of γδ[T2] and γδ[2] cells Cells were co-stained with anti-γδ TCR-FITC, CD27-PE and CD45RA-APC antibodies. Quadrant settings were determined using isotype controls. Note the less differentiated nature of γδ[T2] cells. CM – central memory; EM- effector memory; Term – terminally differentiated cells.

17. Determine the purity of γδ T-cells by flow cytometry:
  a. Transfer a minimum of 5 × 10^4^ γδ[2] and γδ[T2] cells into two separate 5 mL flow cytometry tubes.
  b. Wash each tube with 2 mL FACS buffer (PBS with 0.5 mM EDTA and 1% FCS) and centrifuge at 400 × *g* for 5 min.
  c. Discard supernatant and resuspend the cell pellet in 50 μL PBS.
  d. Add 5 μL anti-human γδ TCR-FITC antibody (BD Biosciences; clone B1; Cat# 559878, RRID: AB_397353 – see key resources table) to one tube containing γδ[2] and γδ[T2] cells.
    ***Note:*** Other anti-human γδ TCR antibodies suitable for flow cytometry are commercially available and may potentially be used for this step.
  e. Add 5 μL FITC-conjugated isotype control to the second tube containing γδ[2] and γδ[T2] cells. Place tubes on ice for 30 min.
  f. Wash each tube with 2 mL FACS buffer and centrifuge at 400 × *g* for 5 min.
  g. Discard supernatant and resuspend cells in 100–250 μL PBS.
  h. Analyze using flow cytometry.
    **Pause Point:** Cells can be maintained in culture for up to day 21. In this case, continue the feeding regimen as described in step 16.
    ***Note:*** Using this method, a median purity of 92.7% γδ T cells is achieved. The lowest purity achieved at which acceptable anti-tumor activity was observed was 74.9%. Flow sorting can be used to improve purity and also to remove contaminating αβ T cells and B cells, which are undesirable impurities in the event that allogeneic γδ T cells are infused for therapeutic purposes.
    ***Note:***Figure 1 presents an example in which γδ[2] and γδ[T2] cells were stained with differentiation markers, CD27 and CD45RA (Dieli et al., 2003). γδ[T2] cultures generally exhibit greater viability and display a less differentiated phenotype (Beatson et al., 2021).
    ***Note:*** The evaluation assays described in the sections that follow are performed independently of each other, rather than representing a sequentially timed protocol.

### Evaluation of *in vitro* real time tumor cytolytic activity of γδ[T2] cells

**Timing: Up to 6 days**

This step describes methods used to quantify the tumor cell killing activity of TGF-β-educated Vγ9Vδ2 T cells using the xCELLigence MP impedance analyzer.

***Note:*** It is recommended to use tumor cell lines that have been passaged 20 times or less.

18. Plate MDA-MB-231 tumor cells (2 × 10^4^ cells each) on a 96 well electronic microtiter plate (Agilent; Cat# 300601010) in 200 μL D10 medium.

***Note:*** DMEM medium is available from many commercial suppliers and may be used for this step.

19. After 24 h, pulse the cells with ZOL (3 μg/mL) or medium alone as a control.
20. After a further 24 h, remove 100 μL medium from each well. Add γδ[T2] and γδ[2] cells in 100 μL TexMACS^TM^+ medium to achieve a final 1:1 E:T ratio.

***Note:*** Add cells gently to minimize disruption to tumor monolayers.

21. Perform dynamic monitoring of adherent tumor viability/ proliferation using an xCELLigence MP impedance analyzer.

***Note:*** This equipment measures cellular impedance (and thereby cellular viability) in a label-free manner. The xCELLigence MP impedance analyzer can host up to six 96-well electronic microplates. The analyzer is maintained in an incubator (37°C, 5% CO_2_).

**Figure 2:**
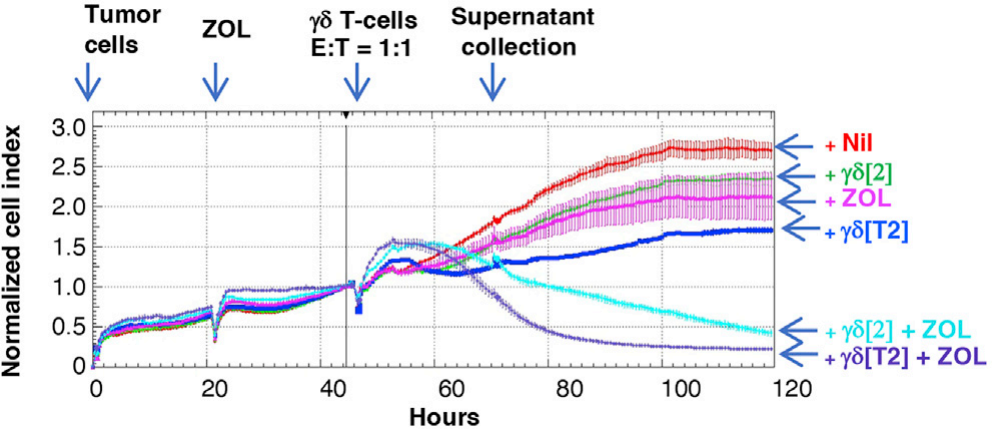
Real time cytolytic activity of γδ[T2] and γδ[2] cells In this example, MDA-MB-231 tumor cells were added to a 96 well electronic microtiter plate. Zoledronic acid (3 μg/mL) and either γδ[2] or γδ[T2] cells were added after 24 and 48 h respectively. Tumor cell number is normalized to the number present at the time of γδ T cell addition (“normalized cell index”).

22. A representative example of the analysis is shown in Figure 2.

### Evaluation of *in vitro* tumor cytolytic activity and cytokine release by γδ[T2] cells

**Timing: 3–5 days**

This step describes methods used to quantify the tumor cell killing activity of TGF-β-educated Vγ9Vδ2 T cells using an MTT assay. Measurement of cytokine release by these cells by ELISA is also described.

***Note:*** It is recommended to use tumor cell lines that have been passaged 20 times or less.

23. Plate tumor cells (1 × 10^5^ cells each) in a 24 well microtiter plate in 1 mL D10 medium (see materials and equipment Table above).
24. After 24 h, pulse tumor cell monolayers with the indicated concentration of ZOL or pamidronic acid (PAM).

***Note:*** These are alternative aminobisphosphonate drugs that cause phosphoantigen accumulation in tumor cells, thereby sensitizing them to γδ T cells.

25. After a further 24 h, remove half of the medium and add γδ[T2] and γδ[2] cells in 500 μL TexMACS^TM^+ medium to achieve a final 5:1 E:T ratio.
26. After a further 24 h, collect 200 μL supernatant from each well for cytokine analysis by ELISA, as described by the manufacturers.

***Note:*** Supernatants can be harvested 24–72 h after addition of γδ T cells. While IFN-γ is readily detectable at these time points, IL-2 levels are much lower and are optimally detected at 24 h after γδ T cell addition.

***Note:*** Supernatants are diluted 1 in 5 to measure IFN-γ and are analyzed undiluted to measure IL-2. Follow ELISA manufacturer’s protocols to measure IFN-γ and IL-2 for this step.

**Figure 3:**
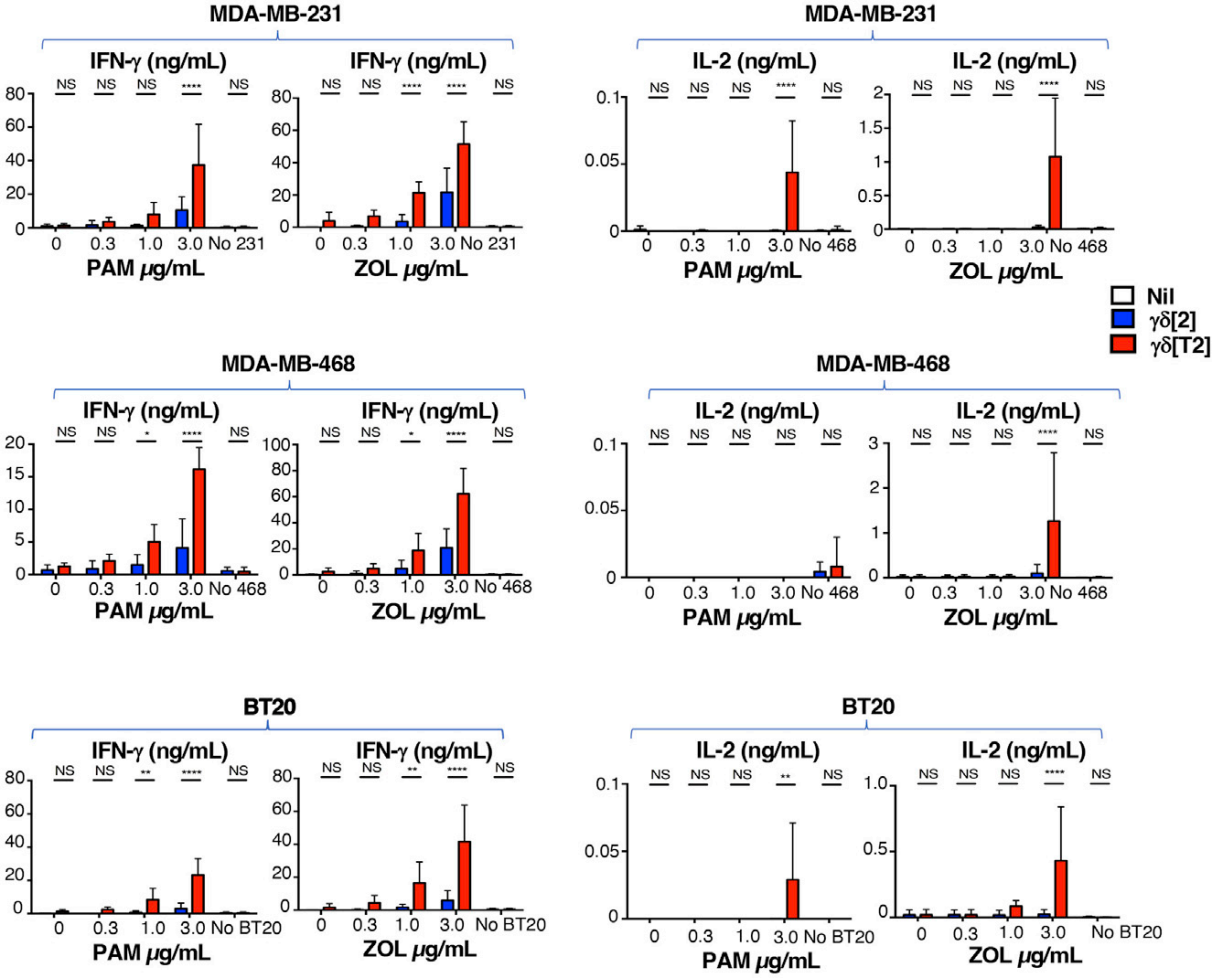
Cytokine release by γδ[T2] and γδ[2] cells γδ[2] cells and γδ[T2] cells were co-cultivated with the indicated triple negative breast cancer monolayers (5:1 E:T ratio) that had been pulsed 24 h earlier with ZOL or PAM at concentrations specified. Release of IFN-γ and IL-2 was measured by ELISA in supernatants collected after 24 h. All data show mean + SD of n=3–5 replicates. All statistical analysis was performed using two-way ANOVA, making comparison between γδ[2] cells and γδ[T2] cells. ∗ p<0.05; ∗∗ p<0.01; ∗∗ p<0.001;∗∗∗∗ p<0.0001; NS – not significant.

***Note:***Figure 3 provides examples of IFN-γ and IL-2 release by γδ[T2] and γδ[2] cells when co-cultivated with a panel of triple negative breast cancer cell lines that had been pre-sensitized with the indicated concentration of PAM or ZOL. Further examples are provided in (Beatson et al., 2021).

27. Seventy two hours after addition of γδ[T2] and γδ[2] cells, assess residual tumor cell viability using the 3-(4,5-Dimethylthiazol-2-Yl)-2,5-Diphenyltetrazolium Bromide (MTT) assay.
  a. Prepare MTT to a concentration of 500 μg/mL in D10 medium (13 mL/24 well plate).
  b. Carefully remove media from each well of one 24 well plate (leaving two plates for 48 h and 72-h analysis).
  c. Pipette 500 μL of 500 μg/mL MTT solution into each well.
  d. Incubate at 37°C & 5% CO_2_ for 1 h.
  e. Aspirate MTT solution from each well being careful not to disturb the monolayers.
  f. Add 500 μL DMSO into each well.
  g. Gently swirl the plate to solubilize the formazan crystals.
  h. Read absorbance at 560 nm using a plate reader.
  i. Calculate residual tumor cell viability using the following equation:

(Absorbanceoftumor+T−cells/absorbanceoftumor+mediumcontrol)x100

**Figure 4:**
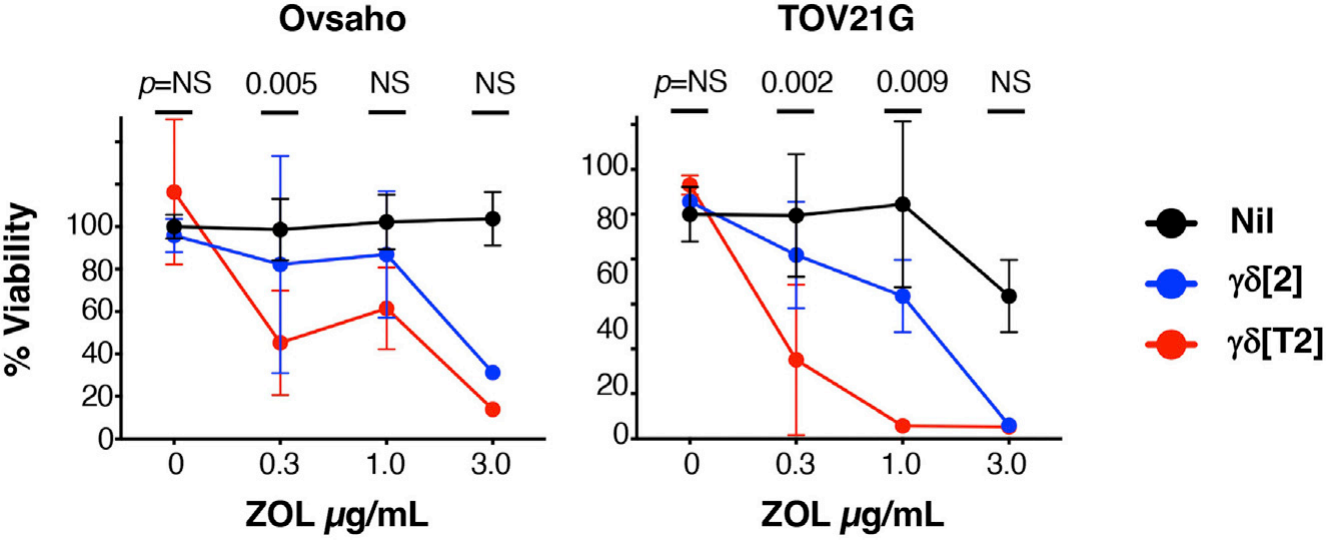
Tumor cytolytic activity of γδ[T2] and γδ[2] cells γδ[2] cells and γδ[T2] cells were co-cultivated with the indicated ovarian cancer monolayers (5:1 E:T ratio) that had been pulsed 24 h earlier with ZOL (at concentrations specified). Black lines represent monolayers without the addition of γδ cells. Residual tumor cell viability was measured after 72 h by MTT assay. All data show mean + SD of n=3–6 replicates. All statistical analysis was performed using two-way ANOVA, making comparison between γδ[2] cells and γδ[T2] cells. NS – not significant.

***Note:***Figure 4 provides examples of tumor cell killing by γδ[T2] and γδ[2] cells when co-cultivated with a panel of ovarian cancer cell lines that had been pre-sensitized with the indicated concentration of PAM or ZOL. Further examples involving triple negative breast cancer cell lines are provided in (Beatson et al., 2021).

### Evaluation of basement membrane invasion by γδ[T2] cells

**Timing: 3 days**

This step describes methods used to quantify the ability of TGF-β-educated Vγ9Vδ2 T cells to invade basement membrane extract.

Day -1.

28. Block the required number of wells in rows B and C of a 24 well plate(s) with 1% BSA in PBS overnight (12–16 h) at 4°C.
29. Place sterile 1.5 mL Eppendorfs, 20 and 200 μL pipette tips at 4°C overnight (12–16 h).

Day 0.

30. Thaw Engelbreth-Holm-Swarm (EHS) mouse sarcoma-based basement membrane extract (BME) on ice.

**CRITICAL:** An icebox should be placed within a tissue culture hood to maintain sterility and low-temperature.

31. Aspirate the blocking solution (see step 1) from the 24 well plate and replace with 650 μL TexMACS^TM^+ medium per well.
32. Mix BME with cold TexMACS^TM^+ media at a 1:1 ratio on ice (within hood) using a cold 1.5 mL Eppendorf and pipette tips (keep on ice).

***Note:*** The volume prepared should allow for coating of the required number of Transwell inserts with 80 μL of the mixture. A small excess should be prepared to allow for losses during pipetting.

33. Coat ThinCert^TM^ inserts (6.5 mm, 3 μm pore size) with 80 μL BME/TexMACS^TM^+ solution using cold pipette tips and placed in rows A and D of the 24 well plate.

**CRITICAL:** Make sure the solution is spread evenly and there are no bubbles. If there are bubbles, very gently use a tip to remove, being careful not to touch the membrane of the insert. If this is not possible, take a new insert and start again.

34. Place the 24 well plate in an incubator (37°C) for at least 30 min or at room temperature (approximately 20°C–22°C) for >1 h to allow the BME to solidify.
35. Remove expanded γδ[T2] and γδ[2] cells from culture into a 15 mL Falcon tube and count live cells using trypan blue and a hemocytometer.

***Note:*** there is no need for trypsin or any disassociation agent as the cells do not attach to plastic.

36. Centrifuge expanded γδ[T2] and γδ[2] cells at 350 × *g* for 5 min at room temperature (approximately 20°C–22°C). Remove supernatant and resuspend the cells at a density of 1 × 10^6^/mL.
37. If antagonism of cell surface proteins is being assessed, treat γδ T cells with an appropriate blocking antibody or control in PBS prior to the start of the assay.

**Figure 5:**
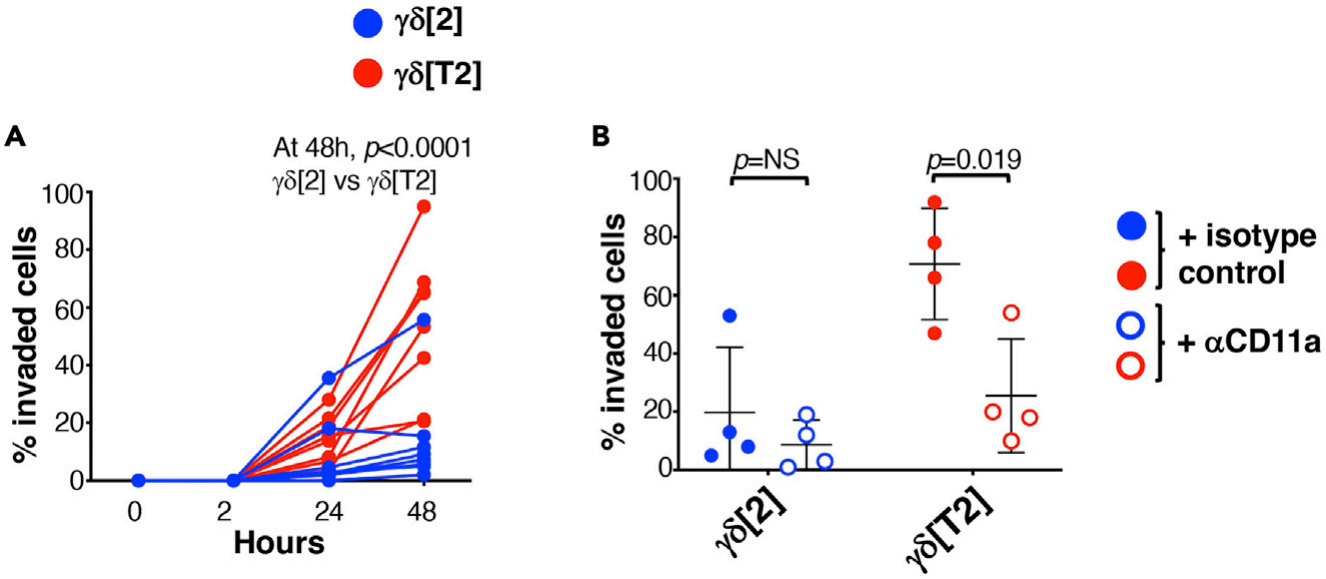
Basement membrane extract invasion by γδ[T2] and γδ[2] cells (A) γδ[2] or γδ[T2] cells were placed on BME within ThinCerts^TM^ which were placed in TexMACS^TM^+. Cells that invaded into the lower well were enumerated at the indicated time points (n=8 per group). (B) The experiment in A was repeated in the presence of a blocking antibody directed against CD11a or an isotype control (mean +/- SD, n=4). Invaded cells (%) were measured at 48 h. Statistical analysis was performed using an unpaired Student’s *t*-test.

***Note:*** Steps 37 or 38 should be performed <5 min before the γδ[T2] and γδ[2] cells are added to coated ThinCert^TM^ inserts in rows A and D.

***Note:*** In the example shown in Figure 5, the effect of CD11a blockade has been tested, making comparison with an isotype control at the same concentration. In this example, γδ T cells are treated with 10 μg/mL of anti-CD11a or isotype control immediately prior to placement of cells in ThinCert^TM^.

38. If migration towards a particular factor or supernatant is being assayed, add the relevant factor to (or substitute for) medium in the appropriate well in rows B-C. This has not been tested in the example shown in Figure 5.

39. Remove the 24 well culture plate from the incubator.
40. Gently add 150 μL of re-suspended γδ[T2] and γδ[2] cells (e.g., 1.5 × 10^5^ cells) to the top of the coated ThinCert^TM^ inserts in rows A and D, thereby placing the cells on top of the BME.
41. The assay commences when these inserts are transferred into the media-containing wells in rows B and C.
42. At predetermined time points, carefully remove the inserts from the media and place these in the corresponding columns in rows A and D of the 24 well plate.
43. Count the number of cells present in the medium using a hemocytometer. It is advisable to take three measurements for each well, as there can be large variations.

***Note:*** A representative example of this protocol is shown in Figure 5. Comparison is made between γδ[T2] and γδ[2] cells. Inhibition of invasion by CD11a blockade has also been evaluated, implicating CD11a in this process.

### Engineering of γδ[T2] T cells to express a chimeric antigen receptor

**Timing: Minimum 2 weeks (to allow for transduction and expansion of the cells)**

This step describes methods used to engineer TGF-β-educated Vγ9Vδ2 T cells to express a chimeric antigen receptor.

***Note:*** For vector design protocols and transient retroviral production protocols using triple transfected 293T cells, please see Larcombe-Young et al. (*currently under review as a Star Protocol*). Using this method, viral titer typically ranges from 5–10 × 10^5^ viral particles/mL. Viral titer is not routinely determined prior to proceeding as described below.

**Figure 6:**
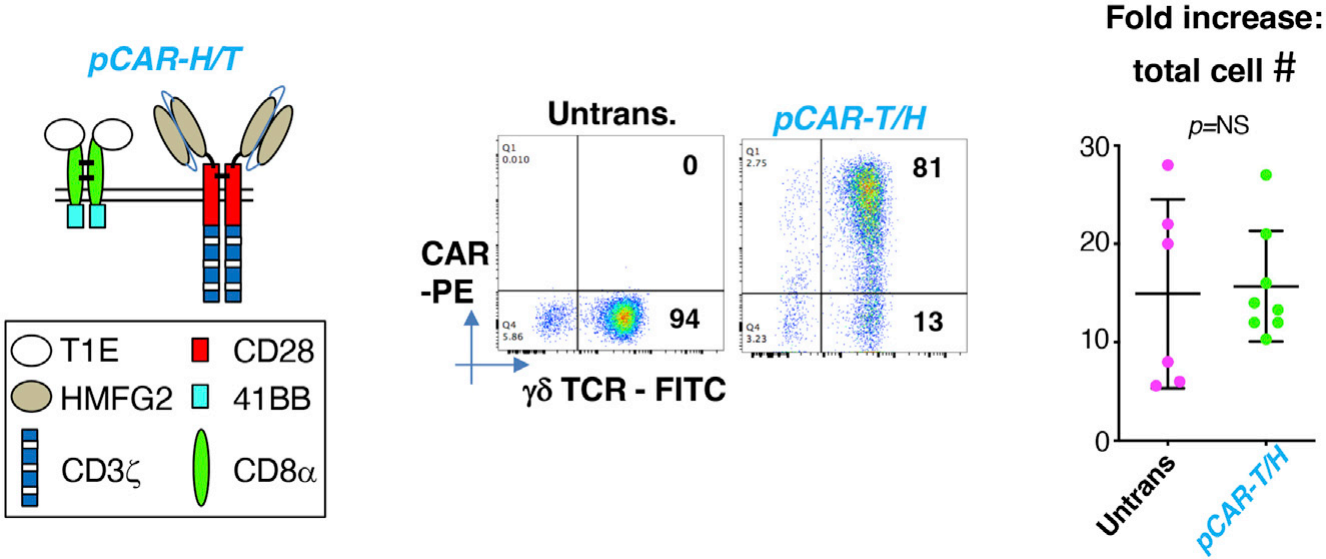
Expression of the *pCAR H/T* parallel CAR in retrovirus-engineered γδ[T2] cells The structure of *pCAR H/T* is shown schematically on the left. A representative example of flow cytometric analysis of γδ[T2] cells that express *pCAR H/T* are shown in the middle. Expansion of *pCAR H/T* γδ[T2] cells is shown on the right, expressed as fold increase in total cell number Note similar expansion efficiency to untrans(duced) γδ[T2] cells.

***Note:*** 1 million cells are transduced in each well of the RetroNectin®-coated 6 well plate. Calculate how many wells are needed for each construct to be transduced.

***Note:*** Parallel (p)CAR is a recently described technology in which a second generation (CD28-containing) CAR is co-expressed with a 4-1BB containing chimeric co-stimulatory receptor. The *pCAR-H/T* parallel CAR co-targets MUC1 and ErbB dimers and is shown in schematic form in Figure 6. Transduced cells are conveniently detected using anti-EGF antibody which binds specifically to one targeting moiety present in *pCAR-H/T*. Further details of the *pCAR-H/T* pCAR are provided in (Muliaditan et al., 2021).

***Note:*** this protocol is optimized for transduction of γδ[T2] cell cultures which have been cultured for 72 h on immobilized pan γδ TCR antibody (11F2 clone).

44. 24 h before transduction:
  a. Add 100 IU/mL recombinant human IL-2 to activated γδ[T2] cells.
  b. Add 200 μL RetroNectin® (1 mg/mL stock solution) to 18 mL sterile PBS in a 50 mL Falcon tube.
  c. Using a Pasteur pipette, aliquot 3 mL into each well of a non-tissue culture-treated 6 well plate.
  d. Wrap the 6 well plate in Saran Wrap and store overnight (12–16 h) in a refrigerator at 4°C.

**CRITICAL:** RetroNectin® can stick to certain plastics. Use polypropylene Pasteur pipettes to minimize protein loss during reagent preparation.

45. On the day of transduction:
  a. Rapidly thaw 1.5 mL aliquots of viral supernatant in a 37°C water bath for the retroviral constructs of choice.
    ***Note:*** A total of 4.5 mL viral supernatant is required per 1 × 10^6^ cells to be transduced, of which 1.5 mL will be used to pre-load the appropriate well of the RetroNectin®-coated 6 well plate.
  b. Spray cryovials containing viral supernatant with 70% ethanol and transfer into a laminar flow hood.
  c. Pre-load the RetroNectin®-coated 6 well plate with viral vector by removal of RetroNectin® from each individual well and prompt addition (to avoid drying) of 1.5 mL of thawed viral supernatant.
  d. Incubate virus coated six well plate for 2 h or overnight (12–16 h) at 4°C.
  e. Retrieve activated γδ[T2] cells from antibody-coated plate and transfer into a Falcon tube. After gentle mixing by inversion, count using a hemocytometer and Trypan blue.
  f. Aspirate the 1.5 mL viral vector from the preloaded RetroNectin®-coated 6 well plate.
  g. Add 3 mL of freshly thawed viral supernatant to each well.
  h. Add 1 × 10^6^ activated γδ[T2] cells per well of the 6 well plate.
  i. For non-transduced control γδ[T2] cells, add 3 mL D10 medium.
  j. Add IL-2 to each well to a final concentration of 100 IU/mL.
  k. Incubate cells at 37°C & 5% CO_2_ for 72 h.
    ***Note:*** Activated T-cells should not be washed prior to counting and transduction as this will remove potentially stimulatory factors present in the medium that they have conditioned.
    ***Note:*** Do not use all activated T-cells for transduction. Untransduced T-cells will later be required, for example, as a control for flow cytometry analysis and/ or functional studies.

46. Every 2 days (3 days if leaving over weekends), feed γδ[T2] cells with 100% volume increase of TexMACS^TM^+ medium, with the addition of recombinant human IL-2 to a final concentration of 100 IU/mL and recombinant human TGFβ1 to 5 ng/mL. Over this interval, maintain T cells at 37°C and 5% CO_2_.

***Note:*** If cell density is below 5 × 10^5^/mL, add 100 IU/mL recombinant human IL-2, and 5 ng/mL recombinant human TGFβ1 only.

47. On day 15 of the culture, determine the transduction efficiency of the γδ T-cells by flow cytometry.
  ***Note:*** The staining procedure is summarized in Table 1.
  a. Transfer 2.5 × 10^5^ transduced and untransduced γδ[T2] cells into three separate 5 mL flow cytometry tubes.
  b. Wash each tube with 2 mL PBS and centrifuge at 400 × *g* for 5 min.
  c. Discard PBS and resuspend the cell pellet in 50 μL PBS.
  d. *Step 1*: Add 5 μL anti-human γδ TCR-FITC antibody to one tube containing untransduced γδ[T2] cells (Ut tube 1).
  e. Add 5 μL FITC-conjugated isotype control to the second tube containing untransduced γδ[T2] cells (Ut tube 2).
  f. Add 5 μL anti-human γδ TCR-FITC antibody to all three tubes containing transduced γδ[T2] cells (Td tubes 1–3).
  g. Place tubes on ice for 30 min.
  h. Wash each tube with 2 mL FACS buffer and centrifuge at 400 × *g* for 5 min.
  i. Discard PBS and resuspend cells in Ut tube 1, Ut tube 2 and Td tube 1 in 250 μL PBS. These tubes are now ready for analysis.
  j. Add 50 μL PBS to Ut tube 3, Td tube 2 and Td tube 3.
  k. *Step 2*: Add 3 μL biotinylated anti-human EGF antibody to Ut tube 3 and Td tube 3 and place on ice for 30 min in the dark.
  l. Wash each tube with 2 mL PBS and centrifuge at 400 × *g* for 5 min.
  m. Discard PBS and add fresh 200 μL PBS to Td tube 2. This tube is now ready for analysis.
  n. Discard PBS and add 50 μL to Ut tube 3 and Td tube 3.
  o. *Step 3*: Add 3 μL Streptavidin-PE to Ut tube 3, Td tube 2 and Td tube 3 and incubate on ice for 30 min in the dark.
  p. Wash each tube with 2 mL PBS and centrifuge at 400 × *g* for 5 min and return to ice.
  q. Discard PBS and resuspend cells in 250 μL PBS.
  r. Analyze using flow cytometry.
    ***Note:*** A representative example of expression of pCAR-H/T in retrovirus transduced γδ[T2] cells is shown in Figure 6, together with efficiency of *in vitro* expansion of these cells and untransduced control cells.

**Table 1:** Staining of CAR expression in transduced γδ[2] and γδ[T2] cells

Tube	Step 1	Step 2	Step 3	Purpose
Ut1	anti-human γδ TCR-FITC			Detect Ut γδ T-cells
Ut2	Isotype FITC			Set baseline FITC signal to detect Ut γδ T-cells
Ut3		Anti-EGF biotin	Streptavidin PE	Set baseline APC signal to detect transduced cells (highest of Ut3 vs Td2)
Td1	anti-human γδ TCR-FITC			Detect γδ T cells in transduced population. Should correlate with number identified in Td2 and Td3 tubes
Td2	anti-human γδ TCR-FITC		Streptavidin PE	Set baseline APC signal to detect transduced cells (highest of Ut3 vs Td2)
Td3	anti-human γδ TCR-FITC	Anti-EGF biotin	Streptavidin PE	Detect transduced γδ T-cells

### Measuring *in vitro* tumor cytolytic activity and re-stimulation potential of γδ[T2] CAR T cells

**Timing: 4 days minimum**

This method quantifies tumor cell killing by pCAR γδ[T2] cells that target MUC1 and in which signaling is boosted by ErbB dimer co-expression, making comparison with untransduced γδ[T2] cells. The method can also be extended to assess tumor re-stimulation potential. This provides a measure of resistance of the CAR-engineered γδ T cells to exhaustion, induced by repeated antigen exposure (Vardhana et al., 2020).

***Note:*** It is recommended to use tumor cell lines that have been passaged 20 times or less.

48. Day 1 – prepare tumor cells:
  a. Seed tumor cells at 1 × 10^5^ cells in 500 μL D10 medium per well in tissue culture treated 24 well plates.
    ***Note:*** Ensure that triplicate wells are plated for each T-cell population to be tested.
  b. Incubate cells at 37°C & 5% CO_2_ for 24 h.
    ***Note:*** validate tumor cell surface expression of the target antigen(s) recognized by the CAR under study prior to co-culture experiments. In this example, firefly luciferase-expressing BxPC3 tumor cells have been used which co-express both MUC1 and multiple ErbB dimers (Whilding et al., 2017) and which have limited intrinsic sensitivity to killing by γδ T-cells.

49. Day 2 – prepare T cells:
  a. Count transduced and untransduced γδ[T2] cells. Re-suspend to a density of 2 × 10^5^ cells per 0.5 mL TexMACS^TM+^ media.
    ***Note:*** This provides cells for a 2:1 E:T ratio. An excess should be prepared so that cells can be processed by serial 2-fold dilution.
  b. Add 0.5 mL CAR or non-transduced γδ[T2] cells per well of tumor cells in triplicate.
  c. Add 1 mL of TexMACS^TM+^ / D10 medium (equal parts mixture) to three wells to act as tumor alone control.
  d. Incubate plate 37°C & 5% CO_2_.
    ***Note:*** No cytokine support is provided for T cells during the co-culture assay.

50. Day 3 (*Optional*) – collect supernatant for cytokine analysis as per step 31.
51. Day 4 – analyze cytotoxicity.
  a. Add d-luciferin at 150 mg/mL immediately prior to luminescence reading.
  b. Measure luminescence using appropriate apparatus and settings.
  c. Calculate tumor cell viability using the following equation:

(luminescenceoftumorcellsculturedwithTcells/luminescenceofuntreatedmonolayeralone)x100%.

52. To assess tumor re-stimulation potential, repeat steps 48–51 twice per week until T cells can no longer be retrieved or less than 25% tumor cell killing occurs.

***Note:*** Donor to donor variation is a significant issue in performing functional studies with primary human CAR T-cells. See troubleshooting section. An adequate number of biological replicates are required to ensure validity of results.

**Figure 7:**
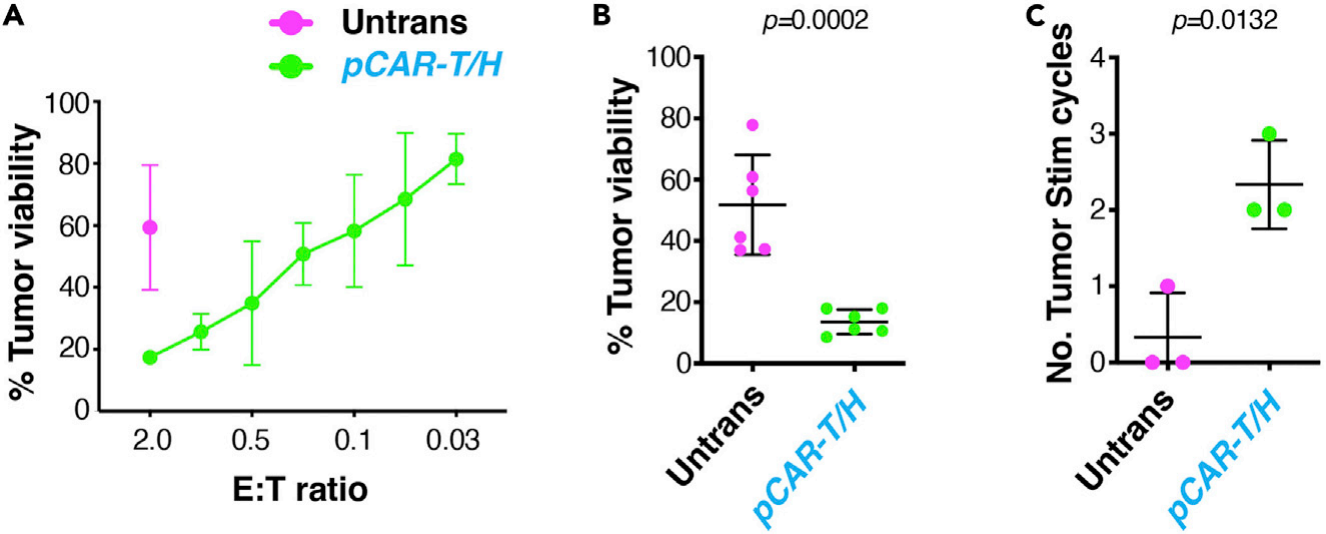
BxPC3 tumor cell killing by *pCAR H/T*-engineered γδ[T2] cells (A) Cytotoxicity assay in which untrans(duced) or *pCAR-H/T* transduced γδ[T2] cells were co-cultured at the indicated effector:target (E:T) ratio with ffLuc^+^ BxPC3 tumor cells for 48 h. Tumor cell viability was determined by luciferase assay (mean +/- SD, n=4). (B) Replicate cytotoxicity assays conducted at a 1:1 E:T ratio for 48 h (mean +/- SD). (C) Firefly luciferase BxPC3 tumor cells were co-cultivated with untrans(duced) or *pCAR-H/T* transduced γδ[T2] cells. Tumor cell viability was measured after 3–4 days. If >25% tumor cell killing occurred, γδ[T2] cells were re-stimulated by transfer to a new tumor cell monolayer. Total number of successful re-stimulation cycles is shown. Statistical analysis was performed using an unpaired Student’s *t* test.

***Note:*** Representative examples of tumor cell cytotoxicity and tumor cell re-stimulation on BxPC3 tumor cells are shown in Figure 7.

### Measuring *in vivo* antitumor activity of γδ[T2] CAR T cells

**Timing: 5–16 weeks**

**Figure 8:**
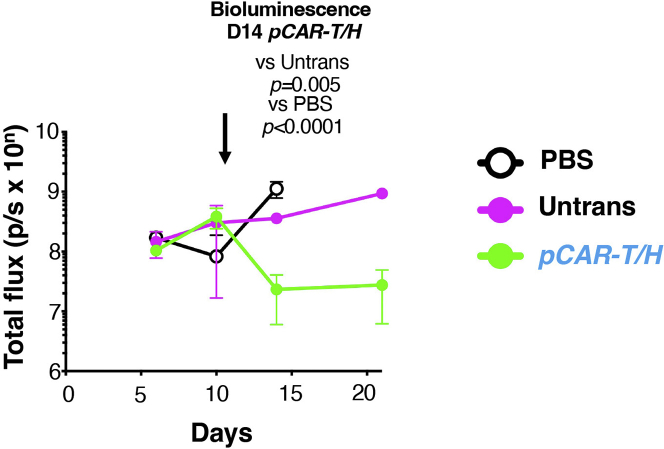
*In vivo* anti-tumor activity of *pCAR H/T*-engineered γδ[T2] cells 1 × 10^5^ ffLuc^+^ BxPC3 tumor cells were injected i.p. in NSG mice. After 12 days, mice were treated with 10 × 10^6^ untrans(duced) or *pCAR-H/T* transduced γδ[T2] cells, making comparison with PBS. Serial BLI emission is shown (mean +/- SD, n=3). Statistical analysis was performed using an unpaired Student’s *t* test.

This method quantifies *in vivo* anti-tumor activity of γδ[T2] cells in a challenging intraperitoneal (i.p.) xenograft model of pancreatic cancer, established using BxPC3 tumor cells. A representative example of such a study is shown in Figure 8. Tumor cells co-express red fluorescent protein (RFP) and firefly luciferase (ffLuc), achieved by retroviral transduction and flow sorting to purity (Whilding et al., 2017). Comparison is made with unmodified γδ[T2] cells that have negligible cytolytic activity against these tumor cells.

***Note:*** It is recommended to use tumor cell lines that have been passaged 20 times or less.

***Note:*** Group sizes should be determined prior to the experiment. In the example shown in Figure 8, a pilot study with 3 mice per group is shown. In definitive studies, a power calculation should be performed. One suitable online tool is found at http://www.biomath.info/power/ttest.htm (accessed February 1^st^, 2022). See troubleshooting step.

***Note:*** To reduce bias, the experiment should be performed in a blinded fashion whereby one team member prepares γδ[T2] cells in coded tubes for administration by a blinded second team member.

***Note:*** All *in vivo* experimentation must comply with local regulatory requirements. The experiment described here was performed using the authority of the U.K. Home Office project license number P23115EBF. The content of that license had been reviewed and approved by the King’s College London animal welfare and ethical review body (AWERB).

53. Day -2 – Isolate and activate PBMCs.
  a. Perform steps 1–17/ 44–47 to expand sufficient numbers of *pCAR-H/T* transduced and untransduced γδ[T2] cells for each treatment group over a 2 week period.

***Note:*** CAR T-cell expansion data from previous experiments is required to calculate the number of γδ[T2] cells required for each group. In general, at least 10 million transduced cells can be generated from 1 million transduced γδ[T2] cells over 10 days when validated viral vector is used for the gene transfer step.

54. 6–10 week-old NSG mice are used for the experiment. In general, equal numbers of male and female mice should be used.

***Note:*** Adequate time must be allowed for mice to acclimatize after delivery from a commercial supplier (generally up to 7 days).

55. Day 1 – Establish BxPC3 human pancreatic ductal adenocarcinoma xenograft in NSG mice.
  a. Take baseline weight readings and check mice for signs of ill health prior to tumor injection.
  b. Inject 1 × 10^5^ ffLuc/RFP-expressing BxPC3 tumor cells suspended in 100 μL sterile PBS into all mice using the i.p. route.
  c. Weigh mice twice weekly and check for signs of ill health daily.

***Note:*** Mice should be humanely killed using an appropriate procedure in the event of weight loss >15%, if total tumor flux exceeds 1 × 10^10^ photons per second (p/s) or if tumor growth impairs normal behavior or vital function. Abdominal distention, dyspnoea, neurological dysfunction, lameness, jaundice, piloerection, hunched posture, inability to groom, inactivity, and/ or inappetence are all additional humane end points and should prompt the humane killing of the affected animal.

56. Day 6 – Perform bioluminescence imaging (BLI) of mice.
  a. Inject mice i.p. with 150 mg/kg D-luciferin.
  b. Place mice into an anesthetic induction chamber (isoflurane 2.5%, with flow rate 2 L/min) until fully anesthetized.
  c. Fifteen minutes after the D-luciferin injection, image the mice with an IVIS Spectrum Imaging platform (Perkin Elmer) with Living Image software, using the auto-exposure function.
  d. Determine total flux (p/s) by creating a region of interest over each entire mouse.
  e. After imaging, return the mice to their cages and monitor until they have fully recovered from anesthesia.
57. Day 11 – Perform bioluminescence imaging of mice.
  a. Mice that have total flux values greater than the enrollment threshold (usually >1 × 10^7^ photons/second) are distributed into treatment groups with similar average tumor burden.
58. Day 12 – Inject the appropriate dose of transduced and untransduced γδ[T2] cells into each mouse using the i.p. route (10 × 10^6^ cells in this example).
59. Monitor the mice daily for signs of ill health.
60. Weigh the animals twice weekly.

***Note:*** Reduced weight, piloerection, ruffled coat, poor appetite, and reduced locomotion can all be signs of graft versus host disease (GvHD). Animals should be humanely killed if they show these signs without improvement for 48 h; if they lose >15% of body weight or if they develop other GvHD related signs such as diarrhea (>48 h).

61. To monitor tumor status, weekly bioluminescence imaging is performed for the duration of the study.
62. Mice with total flux (p/s) values reaching the humane endpoint threshold (1 × 10^10^ p/s) are humanely sacrificed by cervical dislocation or asphyxiation in CO_2_.
63. Tumors are fixed in 10% formalin and paraffin embedded for future analysis.

**CRITICAL:** Prior to starting experiments, follow Perkin Elmer protocols to determine the D-luciferin kinetic curve for your model (https://resources.perkinelmer.com/lab-solutions/resources/docs/SOP_Determine_Luciferin_Kinetic_Curve.pdf, accessed January 2^nd^, 2022). Because bioluminescence emission is tissue dependent, optimal timings between injection of D-luciferin substrate and imaging should be determined prior to starting experiments. Serial imaging is performed every 5 min for a total of 40 min after injection of D-luciferin substrate. Total flux is plotted against time and this curve allows the determination of an imaging time at which maximum total flux is observed (most commonly at 10–20 min post injection).

## Expected outcomes

It is anticipated that γδ[T2] cells will have enhanced viability and yield, accompanied by increased intrinsic anti-tumor cytolytic activity. Activation of γδ[T2] cells is accompanied by increased cytokine production compared to counterparts expanded in IL-2 alone. *In vivo* anti-tumor activity of these cells is also expected to be increased and in a manner that can be targeted using a chimeric antigen receptor (Beatson et al., 2021).

## Quantification and statistical analysis

Statistical significance is determined using GraphPad Prism 9 software. Statistical comparison between two groups is undertaken using an unpaired Student *t*-test, assuming normally distributed data. Comparison of three or more groups is performed using one-way or two-way ANOVA with multiple comparisons, when one or two independent variables were present respectively.

## Limitations

Donor variation, in terms of number, purity and phenotype of *ex vivo* expanded γδ T cells is substantial and there is a need to identify attributes/ biomarkers of the starting material that are predictive of robust expansion of cells with the greatest intrinsic anti-tumor activity.

*In vivo* analysis of human CAR T cell functionality using immunocompromised mice is limited by many factors. Since NSG mice do not have human hematopoietic cells or an intact immune system, it is difficult to predict how CAR T cells might overcome immunosuppressive cellular factors, particularly in the case of solid malignancies. Moreover, in an NSG model, CAR T cells may not be exposed to the same immune suppressive checkpoint molecules, cytokines, and metabolic stresses which may be present in a naturally occurring tumor.

Using healthy donors as a source of PMBCs often results in successful transduction and expansion of CAR γδ[T2] cells. However, in a clinical setting when using autologous patient T cells, prior exposure to treatments such as chemotherapy and radiotherapy may compromise the quality and yield of CAR γδ[T2] cells.

## Troubleshooting

### Problem 1

Low yields of *ex vivo* expanded γδ T cells (steps 1–17).

### Potential solution 1

It is appropriate to check starting material to quantify the number of γδ T cells that are present. Upon culture on immobilized anti-γδ TCR antibody, clustering and enlargement of T cell size are generally visible microscopically. Similar attributes are observed in ZOL-activated cultures after a few days. We have found that freshly isolated PBMC from healthy donors are the most reliable source of these cells. Feeder cells that provide mitogenic and/or co-stimulatory signals to the cells may also be used to enhance cell yield (Xiao et al., 2018).

### Problem 2

Lack of distinctive phenotype associated with γδ[T2] cells (step 17). γδ[T2] cells typically have a highly distinctive phenotype with high expression of CD103, CXCR3, E-selectin binding activity and high levels of cutaneous leukocyte antigen (Beatson et al., 2021).

### Potential solution 2

The quality of the TGFβ1 used to supplement the culture should be checked, if necessary, using a functional assay (Abe et al., 1994). It is also important to closely follow manufacturer’s instructions, especially the addition of hydrochloric acid, when reconstituting this cytokine.

### Problem 3

Tumor engraftment may fail in NSG mice following missed i.p. injection. Similarly, false negative BLI emission values may be obtained for the same reason (see steps 55–63).

### Potential solution 3

Very few tumor cell lines will have 100% engraftment rates. Tumor cells left in PBS for extended periods of time before injection will greatly reduce engraftment rates. It may be useful to inject tumor in 3–4 additional mice to ensure that group sizes are sufficient for meaningful analysis. Tumor growth in PBS-treated mice tends to be most homogeneous meaning that a smaller group size may be tolerated than is the case for T cell-treated mice. If all animals engraft tumor successfully, it is recommended to add additional mice to the key T cell treatment groups. Suspected failed i.p. injection of D-luciferin is generally readily identifiable because of the trend of BLI emission in affected mice. If repeat luciferin injection yields a positive reading, the spurious negative value should be omitted from data analysis.

### Problem 4

Retroviral transduction of γδ[T2] cells may be of sub-optimal efficiency (steps 44–47).

### Potential solution 4

High quality plasmids are required for efficient transfection of HEK293T cells (see Larcombe-Young et al., *currently under review as a STAR Protocol*). HEK293T cells should be of low passage number when transfected. γδ[T2] cells should be activated on the day of gene transfer, indicated by formation of clusters/ clumps of enlarged T cells. If problems persist, it may be appropriate to titrate viral vector in order to ensure that batches contain a high titer of virus.

### Problem 5

Undertaking functional *in vivo* studies using CAR-engineered γδ[T2] cells can be unpredictable due to donor-to-donor variability (see steps 53–63). T cell fitness, state of differentiation and yield can vary significantly between donors.

### Potential solution 5

The primary solution to this issue is to perform each experiment with multiple biological replicates.

### Problem 6

Cytotoxicity assays are conveniently performed using luciferase assays as described in steps 48–51 of this protocol. However, this method requires the stable expression of firefly luciferase in the tumor cells.

### Potential solution 6

If luciferase-expressing tumor cells are unavailable, MTT assays can be performed to quantify cytotoxicity of T cells against adherent tumor cell monolayers. This measures the reduction of tetrazolium in a manner that is dependent on intact mitochondrial function. An example of the use of this assay is provided in steps 23–27.

## Resource availability

### Lead contact

Further information and requests for resources and reagents should be directed to and will be fulfilled by the lead contact Dr John Maher (john.maher@kcl.ac.uk).

### Materials availability

Constructs and other reagents generated or described in this study will be made available from the lead contact for academic/noncommercial research purposes on request. Commercial use of the constructs generated, or derivatives, would be subject to a licensing agreement as intellectual property rights are in place.

## Data Availability

The datasets supporting this protocol, and constructs described or used in Figures, have not been deposited in a public repository but are available from the corresponding author upon request.
